# Cardiovascular disease risk and associated physical activity factors in gastrointestinal cancer survivors

**DOI:** 10.1186/s12889-024-19097-2

**Published:** 2024-06-21

**Authors:** Su Young Kim, Hye Jung Kang, Ki-Hyun Kim, Dong Uk Kim, Seung-Joo Nam, Jae Kook Yang, Dong Kee Jang, Hyuk Soon Choi, Dae Gon Ryu, Jung Wook Lee, Jong Yoon Lee, Sohee Park, Hyun Jung Lee

**Affiliations:** 1 Division of Gastroenterology, Department of Internal Medicine, Yonsei University Wonju College of Medicine, Wonju, Republic of Korea; 2 Department of Health Informatics and Biostatistics, Graduate School of Public Health, Yonsei University, 50-1 Yonsei-Ro, Sodeamun-Gu, Seoul, 03722 Republic of Korea; 3 Department of Internal Medicine, Chonnam National University Hwasun Hospital, Hwasun, Republic of Korea; 4 Department of Internal Medicine, Pusan National University College of Medicine, Busan, Republic of Korea; 5 Department of Internal Medicine, Kangwon National University School of Medicine, Chuncheon, Republic of Korea; 6 Department of Internal Medicine, Soonchunhyang University College of Medicine, Cheonan, Republic of Korea; 7 Department of Internal Medicine, Seoul Metropolitan Government Boramae Medical Center, Seoul National University College of Medicine, Seoul, Republic of Korea; 8 Division of Gastroenterology and Hepatology, Department of Internal Medicine, Institute of Gastrointestinal Medical Instrument Research, Korea University College of Medicine, Seoul, Republic of Korea; 9 Department of Internal Medicine, Pusan National University School of Medicine and Research Institute for Convergence of Biomedical Science and Technology, Pusan National University Yangsan Hospital, Yangsan, Republic of Korea; 10 Department of Internal Medicine, Kosin University Gospel Hospital, Kosin University College of Medicine, Busan, Republic of Korea; 11 Division of Gastroenterology, Department of Internal Medicine, Dong-A University College of Medicine, Busan, 49201 Republic of Korea; 12 Department of Internal Medicine and Liver Research Institute, Seoul National University College of Medicine, 101 Daehak-Ro, Jongno-Gu, Seoul, 03080 Republic of Korea

**Keywords:** Cancer survivors, Gastrointestinal cancer, Atherosclerotic cardiovascular disease, Physical activity

## Abstract

**Introduction:**

Although the risk of CVD is increased in cancer survivors, few studies have investigated the CVD risk in survivors of gastrointestinal (GI) cancer. Therefore, we evaluated the CVD risk using the 10-year atherosclerotic cardiovascular disease (ASCVD) risk score for GI cancer survivors and associated physical activity factors.

**Methods:**

Using the 2014–2019 Korean National Health and Nutrition Examination Surveys, data were collected for 262 GI cancer survivors and 1,310 cancer-free controls matched at a 1:5 ratio based on age and sex. The International Physical Activity Questionnaire Short-Form was used to assess physical activity, and the Euro QoL Questionnaire 5-Dimensional Classification (EQ-5D) was used to assess the health-related quality of life.

**Results:**

A multiple logistic regression analysis demonstrated a lower risk of ASCVD in GI cancer survivors than in controls (adjusted odds ratio [aOR] = 0.73, 95% confidence interval [CI] = 0.55-0.97). Moreover, the risk of having a high ASCVD score was significantly lower in individuals who performed sufficient aerobic physical activity (aOR = 0.59, 95% CI = 0.47-0.75) and those with an EQ-5D score 1 or 2 (aOR = 0.36, 95% CI = 0.20-0.65 and aOR = 0.31, 95% CI = 0.16-0.58, respectively).

**Conclusions:**

This population-based study demonstrated that engaging in sufficient physical activity can reduce the ASCVD risk among GI cancer survivors.

**Supplementary Information:**

The online version contains supplementary material available at 10.1186/s12889-024-19097-2.

## Introduction

With advancements in medical technology and the implementation of cancer screening programs, the overall cancer incidence rate is decreasing and the number of cancer survivors is increasing [1–3]. The increasing number of cancer survivors is accompanied by an increase in non-cancer-related mortality and cancer-related chronic health problems [4]. Gastrointestinal (GI) cancer is one of the most common cancers, with gastric cancer and colorectal cancer (CRC) having a high incidence rate worldwide [5]. Korea has implemented national cancer screening programs to detect GI cancers (gastric cancer and CRC) at an early stage [6]. As a result, the mortality rate from GI cancer has decreased, leading to an increased number of GI cancer survivors and significantly prolonged life expectancy [7, 8]. Consequently, the management of comorbidities among GI cancer survivors has received significant attention.

Cardiovascular disease (CVD) is a significant comorbidity that can increase the mortality risk of cancer survivors [1]. Cancer and CVD share several risk factors and pathophysiological processes that may predispose people to both diseases [9]. Additionally, certain cancer treatments are cardiotoxic and can increase the CVD risk in cancer survivors [10]. The 10-year atherosclerotic cardiovascular disease (ASCVD) risk score proposed by the American College of Cardiology/American Heart Association in 2013 is the most accurate method for assessing the CVD risk [11]. Given the high prevalence of CVD among GI cancer survivors, it is essential to estimate the future risk of ASCVD to provide appropriate preventive and early detection strategies for CVD.

CVD risk can be reduced by engaging in sufficient physical activity. Recent guidelines recommend that adults perform aerobic activities at a certain level or higher [12]. Another guideline recommends that all cancer survivors perform regular exercise for at least 90 min per week [13]. However, it remains unclear how physical activity influences the incidence of CVD in cancer survivors.

Although the CVD risk is increased in cancer survivors, few studies have evaluated the CVD risk in GI cancer survivors [7, 14–17]. Only one study has used the 10-year ASCVD risk score to objectively evaluate the CVD risk in GI cancer survivors [18]. Prior studies only assessed the CVD risk among cancer survivors, and did not investigate the impact of physical activity on the lifestyle of patients. Therefore, we evaluated the 10-year ASCVD risk score for GI cancer survivors and associated physical activity factors.

## Method

### Study population

We used data from the 2014–2019 Korean National Health and Nutrition Examination Surveys (KNHANES) [19]. KNHANES is an annual cross-sectional national survey conducted by the Korea Centers for Disease Control and Prevention. This survey collects data from 25 households from each of the 192 districts, and includes approximately 10,000 individuals annually. The survey gathers information on health examinations, health behavior, and nutrition based on interviews and questionnaires.

**Fig. 1:**
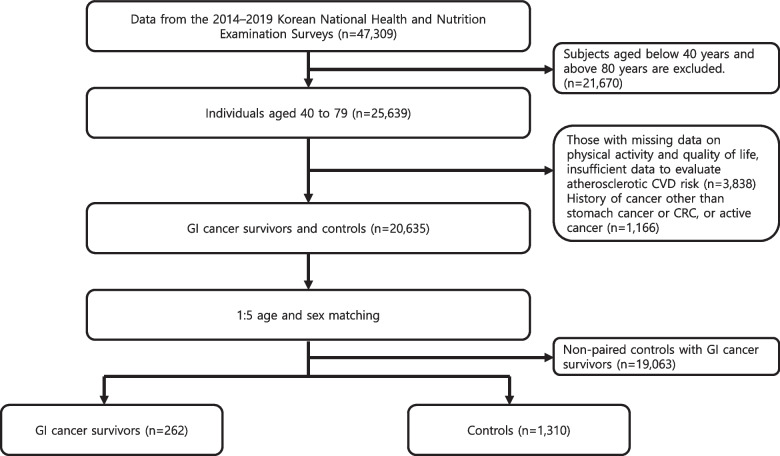
The flow chart of study population

From among the 47,309 participants in the 2014–2019 KNHANES, we selected 25,639 participants aged 40–79 years. Of these participants, those with missing data on physical activity and quality of life, insufficient data to evaluate atherosclerotic CVD risk, history of cancer other than stomach cancer or CRC, or active cancer were excluded. GI cancer survivors were selected based on self-reported responses to the following question: “Have you ever been diagnosed with gastric or colorectal cancer by a doctor?” Controls without cancer were matched to cancer patients according to age and sex at a ratio of 5:1. In total, 262 GI cancer survivors and 1,310 controls were included in the study. Figure 1 presents the participant selection process.

Because the database did not include personal identifiers, the need for informed consent was waived by Center for Human Research Protection of Seoul National University Hospital. Ethical approval was given by the Seoul National University Hospital Institutional Review Board (E-2307–003-1444).

### Assessment of ASCVD risk

ASCVD risk was calculated using the 10-year ASCVD risk score based on the 2013 American College of Cardiology/American Heart Association (ACC/AHA) guidelines [11]. The ASCVD risk estimates were developed based on sex-and race-specific proportional hazards models that included the covariates of age, treated or untreated systolic blood pressure, total cholesterol, high-density lipoprotein cholesterol, current smoking, and diabetes. Individuals with ACC/AHA ASCVD risk > 10% were considered as having a high risk of ASCVD [20].

### Covariates

The KNAHNES data were collected by investigators using self-reported questionnaires, face-to-face interviews, and objective measurements. Health behaviors such as smoking, aerobic physical activity, strength exercises, and quality of life were evaluated using self-reported questionnaire. Comorbidities were recorded using face-to-face interviews, and body mass index (BMI) and blood pressure were determined objectively. In addition, the data for total cholesterol and HDL cholesterol were obtained through blood tests.

Physical activity was assessed using the International Physical Activity Questionnaire Short-Form, which recorded the number of minutes per week spent engaging in vigorous aerobic physical activity, moderate aerobic physical activity, and walking [21]. Each question evaluated all types of activities performed for work, leisure, and transportation that lasted for at least 10 min. Sufficient physical activity was defined as vigorous physical activity for at least 3 days per week for > 20 min per day, or moderate physical activity for at least 5 days per week for > 30 min per day. Participants were also asked about the number of days spent engaging in strength exercise during the past week.

Health-related quality of life was evaluated using the Euro QoL Questionnaire 5-Dimensional Classification (EQ-5D) [22]. It consists of five dimensions (mobility, self-care, usual activities, pain/discomfort, and anxiety/depression) with three response levels for each dimension (no, moderate, or severe problem), which is scored 0 (worst health status) or 1 (best health status). EQ-5D was categorized into five groups based on the scores and weight of each dimension: 1 (EQ-5D = 1), 2 (0.899 ≤ EQ-5D < 1), 3 (0.8 ≤ EQ-5D < 0.899), 4 (0.7 ≤ EQ-5D < 0.8), and 5 (− 0.17 ≤ EQ-5D < 0.7) [23].

### Statistical analysis

Continuous variables are presented as means ± standard deviations, and categorical variables are presented as numbers and percentages. Student’s *t-*test and χ^2^ test were used to compare continuous and categorical variables, respectively. Multiple logistic regression analysis was used to determine the adjusted odd ratios (aORs) for high ASCVD risk (ACC/AHA ASCVD risk > 10%) with a 95% confidence interval (CI). Subgroup analyses were performed to determine the ASCVD risk according to sex and cancer type. Statistical analyses were performed using SAS software (version 7.1; SAS Institute Inc., Cary, NC, USA). *P*-values < 0.05 were considered statistically significant.

## Results

### Baseline characteristics

The baseline characteristics of the GI cancer survivors and controls are presented in Table 1. Sex- and age-matched controls for the GI cancer survivors were selected at a ratio of 5:1. The mean age of the participants was 67.1 ± 8.7 years, and 59.2% were males. In terms of cancer type, 157 (59.9%) had gastric cancer and 105 (40.1%) had CRC, including one individual with both gastric cancer and CRC. The post-cancer survival time of survivors was 11.0 ± 7.5 years. GI cancer survivors had a significantly lower BMI, a lower proportion of current smokers, and a higher level of high-density lipoprotein cholesterol compared to controls. The prevalence of hypertension was also lower among GI cancer survivors than controls. However, there were no significant differences in the levels of sufficient aerobic physical activity, walking, strength exercise, or EQ-5D between GI cancer survivors and controls.

**Table 1:** Baseline characteristics of the study population

Variables	GI cancer survivors (*N* = 262)	Controls (*N* = 1,310)	*P* value
Age (yr)	67.1 ± 8.7	67.1 ± 8.7	0.957
Sex			1.000
Male	155 (59.2%)	775 (59.2%)	
Female	107 (40.8%)	535 (40.8%)	
Cancer type			
Gastric cancer (male/female)	158 (94/64)	-	-
Colorectal cancer (male/female)	105 (61/44)	-	-
BMI (Kg/m^2^)	23.0 ± 3.2	24.2 ± 3.1	< 0.001
Current smoking	30 (11.5%)	232 (17.8%)	0.017
Residence			0.386
Urban	89 (34.0%)	485 (37.0%)	
Rural	173 (66.0%)	825 (63.0%)	
Systolic BP	125.8 ± 17.3	126.0 ± 16.6	0.915
Diastolic BP	74.6 ± 10.2	74.7 ± 9.9	0.753
Hypertension	92 (35.1%)	591 (45.1%)	0.004
Diabetes	55 (21.0%)	289 (22.1%)	0.764
Total cholesterol, mg/dL	186.7 ± 33.6	187.2 ± 39.3	0.817
HDL cholesterol, mg/dL	51.7 ± 13.4	48.3 ± 11.8	< 0.001
Sufficient aerobic physical activity			0.472
No	167 (63.7%)	801 (61.2%)	
Yes	95 (36.3%)	509 (38.9%)	
Walking (per week)			0.354
None	49 (18.7%)	297 (22.7%)	
1–4 days	98 (37.4%)	456 (34.9%)	
5–7 days	115 (43.9%)	557 (42.5%)	
Strength exercise (per week)			0.863
None	205 (78.2%)	1,005 (76.7%)	
1–4 days	31 (11.8%)	164 (12.5%)	
5–7 days	26 (9.9%)	141 (10.8%)	
EQ-5D			0.723
1	155 (59.2%)	762 (58.2%)	
2	28 (10.7%)	145 (11.1%)	
3	44 (16.8%)	208 (15.9%)	
4	24 (9.2%)	111 (8.5%)	
5	11 (4.2%)	84 (6.4%)	

*BMI* Body mass index, *BP* Blood pressure, *HDL* High-density lipoprotein, *EQ-5D* Euro QoL Questionnaire 5-Dimensional Classification

### Associations between GI cancer survivors and ASCVD risk

We determined the aOR for having a high ASCVD risk among GI cancer survivors (Table 2). A multiple logistic regression analysis demonstrated that GI cancer survivors had lower odds ratio of having a high ASCVD risk than controls (aOR = 0.73; 95% CI = 0.55–0.97, *P* = 0.030). Moreover, the odds ratio of having a high ASCVD score were significantly lower in individuals who performed sufficient aerobic physical activity (aOR = 0.59, 95% CI = 0.47–0.75, *P* < 0.001) and had an EQ-5D score 1 or 2 (aOR = 0.36, 95% CI = 0.20–0.65, *P* < 0.001 and aOR = 0.31, 95% CI = 0.16–0.58, *P* < 0.001, respectively). Conversely, the odds ratio of having a high ASCVD score were higher among individuals who performed strength exercise for more than 5 days per week (aOR = 2.15, 95% CI = 1.44–3.20, *P* < 0.001). Additionally, further analysis was conducted based on a five-year cancer survival period that the odds ratio for having high –risk ASCVD were not different across survival periods (Supplementary Table 1).

**Table 2:** Adjusted OR and 95% CI of high-risk of ASCVD (> 10%) in GI cancer survivors

Risk factors	aOR*	95% CI	*P* value
GI cancer survivors	0.73	0.55–0.97	0.030
BMI (Kg/m^2^)	1.00	0.97–1.04	0.986
Urban residence	0.88	0.70–1.10	0.249
Sufficient aerobic physical activity			
No	1		
Yes	0.59	0.47–0.75	< 0.001
Walking (per week)			
None	1		
1–4 days	0.84	0.61–1.15	0.279
5–7 days	0.86	0.62–1.18	0.350
Strength exercise (per week)			
None	1		
1–4 days	1.03	0.74–1.43	0.866
5–7 days	2.15	1.44–3.20	< 0.001
EQ-5D			
1	0.36	0.20–0.65	< 0.001
2	0.31	0.16–0.58	< 0.001
3	0.58	0.31–1.08	0.086
4	0.79	0.39–1.59	0.502
5	1		

*BMI* Body mass index, *EQ-5D* Euro QoL Questionnaire 5-Dimensional Classification

^*^Multivariable adjusted for BMI, residence, physical activity, walking, strength exercise, EQ-5D

### Subgroup analyses and ASCVD risk

Lower odds ratio of having high-risk ASCVD were found among male GI cancer survivors, but not among female survivors (aOR = 0.60, 95% CI = 0.40–0.91, *P* = 0.016 and aOR = 0.84, 95% CI = 0.54–1.31, *P* = 0.445, respectively) (Table 3). In addition, sufficient aerobic physical activity reduced the odds ratio of having a high ASCVD risk in male survivors (aOR = 0.48, 95% CI = 0.34–0.69, *P* < 0.001). Furthermore, sufficient aerobic physical activity (aOR = 0.54, 95% CI = 0.37–0.78, *P* = 0.001), adequate strength exercise for 1–4 days per week (aOR = 0.48, 95% CI = 0.25–0.93, *P* = 0.030), and EQ-5D scores 1–3 (aOR = 0.20, 95% CI = 0.10–0.41, *P* < 0.001; aOR = 0.17, 95% CI = 0.08–0.39, *P* < 0.001; and aOR = 0.41, 95% CI = 0.19–0.89, *P* = 0.025, respectively) were associated with lower odds ratio of having a high ASCVD score in female survivors.

**Table 3:** Comparison of OR and 95% CI of high-risk of ASCVD (> 10%) according to sex

Risk factors	Male			Female		
	aOR*	95% CI	*P* value	aOR*	95% CI	*P* value
GI cancer survivors	0.60	0.40–0.91	0.016	0.84	0.54–1.31	0.445
BMI (Kg/m^2^)	0.96	0.91–1.02	0.167	1.04	0.98–1.09	0.181
Urban residence	0.91	0.65–1.27	0.572	0.8	0.57–1.14	0.212
Sufficient aerobic physical activity						
No	1			1		
Yes	0.48	0.34–0.69	< 0.001	0.54	0.37–0.78	0.001
Walking (per week)						
None	1			1		
1–4 days	0.68	0.41–1.12	0.130	1.00	0.63–1.57	0.984
5–7 days	0.91	0.54–1.55	0.738	0.95	0.60–1.50	0.821
Strength exercise (per week)						
None	1			1		
1–4 days	0.94	0.61–1.47	0.797	0.48	0.25–0.93	0.030
5–7 days	1.46	0.88–2.40	0.142	1.19	0.52–2.73	0.683
EQ-5D						
1	0.43	0.15–1.25	0.120	0.20	0.10–0.41	< 0.001
2	0.49	0.15–1.57	0.229	0.17	0.08–0.39	< 0.001
3	0.74	0.23–2.35	0.608	0.41	0.19–0.89	0.025
4	1.09	0.29–4.07	0.902	0.61	0.26–1.45	0.263
5	1			1		

*BMI* Body mass index, *EQ-5D* Euro QoL Questionnaire 5-Dimensional Classification

^*^Multivariable adjusted for BMI, residence, physical activity, walking, strength exercise, EQ-5D

The odds ratio of having a high ASCVD risk were analyzed by cancer type (Table 4). Gastric cancer survivors showed lower odds ratio for having a high ASCVD risk than CRC survivors (aOR = 0.65, 95% CI = 0.44–0.96, *P* = 0.029 and aOR = 0.81, 95% CI = 0.52–1.27, *P* = 0.362, respectively). Sufficient aerobic physical activity and a lower EQ-5D score were associated with reduced odds ratio of having a high ASCVD score, and excessive strength exercise increased the odds ratio of having a high ASCVD risk regardless of the cancer type.

**Table 4:** Comparison of OR and 95% CI of high-risk of ASCVD (> 10%) stratified by type of cancer

Risk factors	Gastric cancer			Colorectal cancer		
	aOR*	95% CI	*P* value	aOR*	95% CI	*P* value
GI cancer survivors	0.65	0.44–0.96	0.029	0.81	0.52–1.27	0.362
BMI (Kg/m^2^)	1.04	0.99–1.09	0.149	0.97	0.92–1.03	0.267
Urban residence	0.78	0.58–1.05	0.106	0.75	0.53–1.05	0.096
Sufficient aerobic physical activity						
No	1			1		
Yes	0.63	0.46–0.87	0.005	0.63	0.43–0.91	0.014
Walking (per week)						
None	1			1		
1–4 days	1.21	0.80–1.82	0.370	0.62	0.36–1.06	0.081
5–7 days	1.27	0.84–1.92	0.252	0.71	0.41–1.23	0.215
Strength exercise (per week)						
None	1			1		
1–4 days	0.70	0.45–1.08	0.106	1.20	0.70–2.05	0.513
5–7 days	1.78	1.07–2.95	0.026	2.85	1.49–5.43	0.002
EQ-5D						
1	0.22	0.08–0.57	0.002	0.41	0.17–1.00	0.049
2	0.23	0.09–0.64	0.005	0.29	0.11–0.78	0.014
3	0.59	0.21–1.66	0.316	0.74	0.28–1.92	0.535
4	0.43	0.15–1.26	0.124	0.60	0.20–1.74	0.342
5	1			1		

*BMI* Body mass index, *EQ-5D* Euro QoL Questionnaire 5-Dimensional Classification

^*^Multivariable adjusted for BMI, residence, physical activity, walking, strength exercise, EQ-5D

## Discussion

To the best of our knowledge, this is the first study to explore the association between physical activity and ASCVD risk among GI cancer survivors. In this population-based study, the CVD risk was lower in GI cancer survivors than controls. In particular, the odds of a high ASCVD risk were decreased by 41% when adequate aerobic physical activity was performed and was lower for individuals with a better EQ-5D, an indicator of health-related quality of life. Conversely, excessive strength exercise increased the odds of a high ASCVD risk in GI cancer survivors. KNHANES is a government-administered survey with reliable data and comprehensive information on physical activity and quality of life indicators. These data accurately reflect the physical activity of patients in the real world. Our findings based on the KHNANES data are applicable to the real world and have important implications for the behavior of patients.

Several factors can explain the lower ASCVD risk among GI cancer survivors than the general population. First, due to the differences in baseline characteristics, GI cancer survivors had lower oral food consumption compared to the general population, leading to a lower prevalence of obesity and overweight. Surgical intervention for gastric cancer is associated with outcomes comparable to those of bariatric surgery, leading to weight loss and consequently a lower prevalence of metabolic syndrome. Metabolic syndrome is associated with an increased risk of cardiovascular complications [7]. In our findings, the BMI of GI cancer survivors was lower than that of controls. Among all GI cancer survivors, a reduced ASCVD risk was particularly notable in gastric cancer survivors. Second, cancer survivors are likely to adopt a healthier lifestyle after being diagnosed with cancer, including smoking cessation, adherence to prescribed medications for underlying diseases, and engaging in health-promoting habits [24–26]. These behaviors can improve blood pressure, lipid metabolism, and the blood glucose level, thereby reducing the cardiovascular risk. In fact, the current smoking rate was lower and the rates of improved control of blood pressure and HDL cholesterol level were higher in GI cancer survivors than in controls.

Recent studies have shown that physical activity has beneficial effects among cancer survivors, particularly in terms of lowering the CVD risk [27–30]. However, most of the previous studies were conducted on breast cancer patients. To date, no studies have evaluated the CVD risk among GI cancer patients. Jeong et al. evaluated the effects of physical activity on the stroke risk in CRC survivors in 2022, but did not evaluate the CVD risk [31]. In the present study, sufficient aerobic physical activity in GI cancer survivors reduced the odds of having a high ASCVD risk, by 41%. In particular, engaging in strength exercise for 1–4 days per week can effectively decrease the odds of having a high ASCVD risk in women. To improve cancer-related health outcomes, the 2019 physical activity guidelines for cancer survivors recommend 30 min of physical activity per session, three times per week. This time requirement for physical activity is half of that recommended for the general population [13]. EQ-5D evaluates health status and has recently emerged as an important tool for determining prognosis and treatment effectiveness in cancer survivors [32–34]. To date, no studies have evaluated the association between the EQ-5D score and CVD risk in cancer survivors. In the present study, we demonstrated for the first time that the ASCVD risk decreases with decreasing EQ-5D scores. An optimal EQ-5D is associated with as much as 64% lower CVD risk.

Our results demonstrate that excessive strength exercise (5–7 days per week) increased the ASCVD risk in cancer survivors. A recent study of the stroke risk among CRC survivors revealed that higher levels of physical activity were less beneficial than moderate levels [31]. Excessive exercise can alter the function of the sinus and atrioventricular nodes, thereby predisposing patients to atrial fibrillation [35]. Another study demonstrated that long-term vigorous exercise can cause abnormalities of the sympathetic nervous system and atrial enlargement, which are also associated with the development of atrial fibrillation [36]. Furthermore, excessive exercise can accelerate calcium deposition in the coronary artery [37]. Finally, cancer treatments, such as chemotherapy and radiotherapy, can worsen cardiac function. The drug 5-fluorouracil, which is used to treat CRC, is associated with cardiotoxicity [38]. Changes in the cardiovascular system, including heart failure, can occur when such medications are administered, which may reduce the cardiac tolerance of excessive exercise and increase the incidence of adverse effects [39]. In fact, our results demonstrate that a high frequency of strength exercise (5–7 days per week) is associated with a greater increase in the ASCVD risk in CRC survivors than in gastric cancer survivors.

There were several limitations to our study. First, because this was a retrospective study based on data obtained from KNHANES, detailed information regarding the cancer stage and treatment was unavailable. Second, recall bias may have occurred because the frequency and intensity of physical activity were derived from self-reported questionnaires. Finally, the ASCVD risk applied in this study is based on the ACC/AHA guidelines, which primarily target Americans. Our data were obtained from the Korean population, in which the ASCVD risk is lower than that of Western population. Therefore, the precision of risk assessment may be slightly reduced in our study involving Korean subjects. Further comparative studies that include populations with various races are needed.

This population-based study demonstrated that engaging in suitable physical activity can effectively reduce the ASCVD risk among GI cancer survivors. In addition, the EQ-5D score, which assesses quality of life, was inversely proportional to the CVD risk. Our results provide evidence that suitable physical activity can reduce the ASCVD risk among GI cancer survivors, resulting in enhanced long-term survival rates and quality of life.

### Supplementary Information

Supplementary Material 1.

## Data Availability

The datasets generated and analysed during the current study are available in the Korean National Health and Nutrition Examination Surveys repository [https://knhanes.kdca.go.kr/knhanes/eng/index.do]. The datasets generated and/or analysed during the current study are not publicly available but are available from the corresponding author on reasonable request.
