# Supplementary material for: Cardiovascular disease risk and associated physical activity factors in gastrointestinal cancer survivors
Source: BMC Public Health. 2024 Jun 21;24:1656. doi: 10.1186/s12889-024-19097-2 (PMC11191341; doi:10.1186/s12889-024-19097-2)
Supplement: Supplementary file 1 — Supplementary Material 1. [file 12889_2024_19097_MOESM1_ESM.docx]

**Supplementary table 1. Comparison of OR and 95% CI of high-risk of ASCVD (> 10%) according to survival periods**

| Risk factors | Survival period < 5 years | | | Survival period ≥ 5 years | | |
| --- | --- | --- | --- | --- | --- | --- |
|  | aOR* | 95% CI | P value | aOR* | 95% CI | P value |
| GI cancer survivors | 0.61 | 0.27-1.35 | 0.218 | 0.75 | 0.55-1.02 | 0.070 |
| BMI (Kg/㎡) | 0.99 | 0.89-1.11 | 0.910 | 1.00 | 0.96-1.04 | 0.908 |
| Urban residence | 1.01 | 0.53-1.93 | 0.972 | 0.86 | 0.67-1.09 | 0.211 |
| Sufficient aerobic physical activity |  |  |  |  |  |  |
| No | 1 |  |  | 1 |  |  |
| Yes | 0.50 | 0.25-0.96 | 0.039 | 0.62 | 0.48-0.80 | <0.001 |
| Walking (per week) |  |  |  |  |  |  |
| None | 1 |  |  | 1 |  |  |
| 1-4 days | 0.45 | 0.16-1.30 | 0.138 | 0.89 | 0.64-1.24 | 0.485 |
| 5-7 days | 0.70 | 0.24-2.07 | 0.517 | 0.86 | 0.61-1.20 | 0.372 |
| Strength exercise (per week) |  |  |  |  |  |  |
| None | 1 |  |  | 1 |  |  |
| 1-4 days | 3.50 | 1.21-10.19 | 0.021 | 0.85 | 0.60-1.21 | 0.375 |
| 5-7 days | 1.84 | 0.68-4.97 | 0.231 | 2.20 | 1.42-3.42 | <0.001 |
| EQ-5D |  |  |  |  |  |  |
| 1 | 0.32 | 0.10-1.00 | 0.049 | 0.41 | 0.23-0.73 | 0.002 |
| 2 | 0.88 | 0.16-4.74 | 0.884 | 0.31 | 0.16-0.59 | <0.001 |
| 3 | 0.37 | 0.10-1.38 | 0.139 | 0.69 | 0.36-1.31 | 0.257 |
| 4 | 1** |  |  | 0.87 | 0.42-1.81 | 0.703 |
| 5 | - | - | - | 1 |  |  |

BMI, body mass index; EQ-5D, Euro QoL Questionnaire 5-Dimensional Classification

*Multivariable adjusted for BMI, residence, physical activity, walking, strength exercise, EQ-5D

** For subjects with a survival period of less than 5 years, the number of patients in the EQ-5D index '5 (-0.17≤EQ-5D<0.7)' category is zero. Therefore, we have combined this category with index '4 (0.7≤EQ-5D<0.8)' to denote it as '4 (-0.17≤EQ-5D<0.8).
